# Ectopic expression of LAG‐3 in non–small‐cell lung cancer cells and its clinical significance

**DOI:** 10.1002/jcla.23244

**Published:** 2020-02-20

**Authors:** Chenglong Ma, Xiao Sun, Dong Shen, Yuejun Sun, Naifu Guan, Chunjian Qi

**Affiliations:** ^1^ Medical Research Center Changzhou No.2 People's Hospital The Affiliated Hospital of Nanjing Medical University Changzhou China; ^2^ Department of Oncology The Jiangyin Clinical College of Xuzhou Medical University Jiangyin China; ^3^ Department of Pathology The Jiangyin Clinical College of Xuzhou Medical University Jiangyin China; ^4^ Department of Pathology Changzhou No.2 People's Hospital The Affiliated Hospital of Nanjing Medical University Changzhou China

**Keywords:** ectopic expression, LAG‐3, non–small‐cell lung cancer, tumor immunity

## Abstract

**Background:**

Lymphocyte activation gene 3 (LAG‐3, also known as CD223) is an immune checkpoint molecule expressed on various types of lymphocytes, and it is mainly involved in maintaining immune homeostasis. However, there are currently no data on LAG‐3 expression in non–small‐cell lung cancer cells.

**Methods:**

Human lung cancer cells were cultured using conventional methods. The expression of LAG‐3 was measured by Western blot and flow cytometry. Between April 2018 and May 2019, we collected 52 surgical specimens of stage I‐III non–small‐cell lung cancer (NSCLC). Fourteen samples of benign lung tissue lesions were collected as the control group, and the expression levels of LAG‐3 in the lung cancer cells and tissue samples were measured via immunohistochemistry.

**Results:**

Western blots showed that LAG‐3 was expressed in lung cancer cell lines. There was significant difference in the LAG‐3 expression levels in the NSCLC cells and benign lung tissue (*χ* ^2^ = 13.055, *P* = .0003). The LAG‐3 expression level was significantly associated with the NSCLC clinical stage, and LAG‐3 expression was significantly higher in stage III patients (*P* < .05).

**Conclusion:**

LAG‐3 is expressed in NSCLC tumor cells. Furthermore, LAG‐3 not only is expressed in tumor‐infiltrating lymphocytes in NSCLC patients but also is ectopically expressed in tumor cells and associated with TNM stage.

## 1 INTRODUCTION

Lung cancer is the most common malignant tumor in the world and is the leading cause of cancer‐related deaths.^1^ More than 85% of lung cancer patients diagnosed with non–small‐cell lung cancer (NSCLC) have a 5‐year survival rate as low as 15.9%, which has only slightly improved over the past few decades.^2^ Due to improvements in many technologies, including next‐generation sequencing (NGS) and multi‐gene lung cancer mouse models, and the establishment of human tumor databases, our understanding of lung cancer has developed from histopathology to the analysis of the precise molecular characteristics and genotypes of cancer cells. Currently, the main treatment methods for NSCLC include surgery, radiotherapy, chemotherapy, targeted therapy, among others. Although these conventional therapies have achieved some success, the prognosis of NSCLC patients has not improved significantly. Since the development of tumor immunology, tumor immunotherapy has become a new treatment method. The latest immunotherapy methods aim to target immune tolerance mechanisms by blocking immune checkpoints, thus reversing the functional suppression of the immune response, reactivating T cells, and promoting anti‐tumor immunity. In the past few years, therapies targeting the CTLA‐4 and PD‐1 inhibitory receptors have shown clinical efficacy in lung cancer patients. In addition to CTLA‐4 and PD‐1, there are many other immune checkpoint molecules, including TIM‐3, LAG‐3, and TIGIT,^3^ which provide alternative targets that could be utilized to induce an anti‐tumor immune response.

Lymphocyte activation gene 3 (LAG‐3, also known as CD223) is a type I transmembrane protein consisting of 498 amino acids. LAG‐3 is an immune checkpoint molecule expressed on various types of lymphocytes, and it is mainly involved in maintaining immune homeostasis; furthermore, LAG‐3 can play a synergistic role with other immune checkpoint molecules.^4^ LAG‐3 is located on chromosome 12 (12p13.32) adjacent to the CD4 gene. Approximately 20% of its sequence is homologous with that of CD4, and it mainly binds to the major histocompatibility complex (MHCII) molecule on antigen‐presenting cells (APC), promoting cell apoptosis, and limiting proliferation.^5^ LAG‐3 has been shown to bind to four ligands: MHCII,^6^ LSECtin,^7^ galectin‐3,^8^ and fibrinogen‐like protein 1 (FGL1); however, its main inhibitory ligand is FGL1, which can induce T‐cell inhibition and promote immune evasion by blocking the FGL1‐LAG‐3 pathway.^9^ LAG‐3 is involved in the inhibition of T‐cell function, which can lead to the failure of T cells, while synergistic blocking of LAG‐3 and PD‐1 can restore the function of T cells.^10^ Combination immunotherapy with an anti‐LAG‐3 antibody and an anti‐PD‐1 antibody can stimulate a tumor‐specific response, which includes an increased number of effector T cells^11^; furthermore, this treatment is less toxic than single antibody treatment and can improve prognosis.^12^ Therefore, LAG‐3 is not only a valuable marker for evaluating prognosis, and it is also a promising therapeutic target. To strengthen its usefulness in these respects, we measured the expression of LAG‐3 in lung cancer and further analyzed its clinical significance.

## 2 MATERIALS AND METHODS

### 2.1 Cell culture

Human lung squamous cell carcinoma H226 cells, human lung adenocarcinoma H1299, A549 cells, and human lung epithelial cell BEAS‐2B were stored by the Central Laboratory of Changzhou Second People's Hospital, which is affiliated with Nanjing Medical University. The cells were routinely cultured in RPMI 1640 medium containing 10% fetal bovine serum and grown in an incubator at 37°C and 5% CO_2_.

### 2.2 LAG‐3 protein level detection

Western blotting was used to measure the LAG‐3 protein levels in NSCLC cells. Protein was extracted from cells, and its concentration was measured. SDS‐PAGE protein loading buffer (5×) was added to the protein samples, and the mixture was denatured at 100°C for 5 minutes. The protein samples were subjected to SDS‐PAGE electrophoresis, and the proteins were then transferred to a PVDF membrane by a wet transfer method. The membranes were blocked in a solution containing skim milk powder for 1.5 hours, washed 3 times with PBST, and then incubated with mouse anti‐LAG‐3 antibody (LAG‐3, 5 μg/mL, 11E3, ab40465, Abcam) overnight at 4°C with shaking. On the second day, the membranes were washed 3 times with PBST. Next, the corresponding secondary antibody was added, and the membranes were incubated at room temperature with shaking for 1.5 hours. Finally, the membranes were washed and imaged with an instrument.

### 2.3 LAG‐3 expression measurement

The expression of LAG‐3 in NSCLC cells was measured by flow cytometry. After the collected cells were washed with PBS, membrane and intracellular staining were performed according to the manufacturer's instructions. Accordingly, the membrane was stained by adding fluorophore‐labeled anti‐LAG‐3 antibody (PE‐Cy7, Invitrogen), followed by incubation in a dark oven at 4°C for 20 minutes. After the incubation, the cells were washed twice with PBS. For the intracellular staining, the cells were fixed, and then, membrane‐penetrating solution was added. The cells were washed in membrane‐penetrating solution 2 times. After incubation for 10 minutes, the antibody was added, and the cells were incubated at room temperature for 20 minutes in the dark. The cells were then washed twice with PBS. Finally, flow cytometry was used for analysis.

### 2.4 Immunohistochemical examination

#### 2.4.1 Specimen source

Fifty‐two surgical specimens of stage I‐III NSCLC were collected in the Department of Pathology of Jiangyin People's Hospital from April 2018 to May 2019. Lymph node surgical specimens were collected from 11 patients with lymph node metastasis, and samples of 14 benign lesions were also collected. Lung tissue specimens were used as controls. Inclusion criteria were as follows: 1. The patients did not receive radiotherapy or chemotherapy, and 2. the patients had no other malignant tumors. The patients were staged according to the eighth edition of the International Anti‐Cancer Alliance TNM Staging.^13^ All specimens were fixed in 4% formaldehyde solution, embedded in paraffin, and then cut into 4‐mm‐thick sections. Two specimens were cut for each specimen, and they used as a positive control and a blank control.

#### 2.4.2 Immunohistochemistry

Tissue sections were incubated in an oven at 70°C, dewaxed in xylene and ethanol, and then places into citrate buffer (pH 6.0) for antigen retrieval by heating with an induction heat source. Primary antibody (LAG‐3, 1:100, 11E3, ab40465, Abcam) was added, and the samples were incubated overnight at 4°C in a humidified box. Next, secondary antibody (Ultra View Universal HRP Multimer, Ventana) corresponding to the primary antibody was added, and the samples were incubated at 37°C for 30 minutes. DAB color development, which involves soaking in 1% hydrochloric acid to induce lithium carbonate to transition from back to blue, was performed after ethanol gradient dehydration, xylene dehydration, oven drying, and application of a neutral gum seal. Hematoxylin counterstaining was also performed.

The two senior pathologists independently scored the samples under a double‐blind method according to the degree of staining: The cells were essentially not colored, (−); the percentage of positive cell staining was ≤10% or the cell staining was weakly positive but the percentage was ≤30%, (+); the cell staining intensity was moderate to strongly positive, the percentage of positive staining was 10%‐30%, or the staining intensity was weak to moderate, but the percentage of positive staining was between 30% and 50%, (++); and the cell staining was strong in ≥30% of the cells or the cell staining was positive in ≥50% of the cells, (+++).^14^ To determine whether LAG‐3 expression is associated with clinicopathological features, all of the NSCLC patients were divided into LAG‐3 expression‐positive and ‐negative groups. NSCLC patients with LAG‐3 staining intensity scores of (−) and (+) were assigned to the LAG‐3‐negative group, and NSCLC patients with LAG‐3 staining intensity scores of (++) and (+++) were defined as LAG‐3‐positive.

#### 2.4.3 Statistical processing

The flow data were analyzed by the FlowJo10.2 software, and the statistical analysis was performed with SPSS 22.0 software. The count data are expressed by case (%), and intergroup comparison was performed via the chi‐squared test. *P* < .05 was considered significant.

## 3 RESULTS

### 3.1 A survival curve generated based on the relevant data on lung cancer LAG‐3 expression levels in the TCGA database

**Figure 1:**
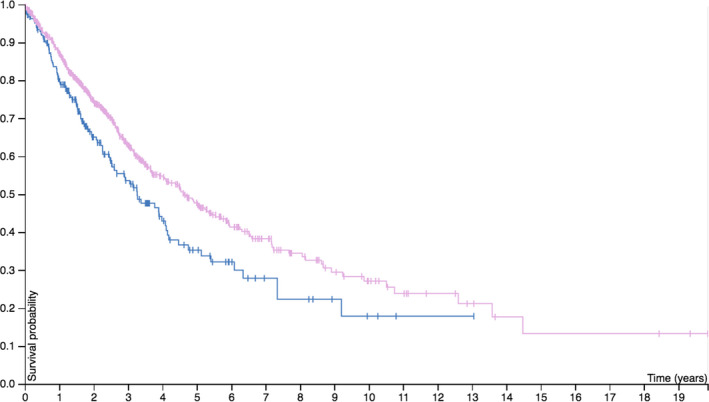
Relationship between LAG‐3 expression and survival rate in patients with lung cancer (data from TCGA database)

The data on LAG‐3 expression in lung cancer in TCGA database were collected, and the relationship between LAG‐3 expression and survival was analyzed to generate a survival curve. The TCGA database has a total of 994 lung cancer patients (ending 2019‐08‐12), including 600 currently living patients, 394 deceased patients, 398 females, and 596 males. There were 219 stage Ia cases, 283 stage Ib cases, 114 stage IIa cases, 159 stage IIb cases, 132 stage IIIa cases, 28 stage IIIb cases, and 32 stage IV cases. There were also patients who could not be staged. Based on an analysis of the LAG‐3 mRNA levels, the 5‐year survival rate was 48% in the LAG‐3 high‐expression group (780 cases), while the low‐expression group (214 cases) had a 5‐year survival rate of 35%. Patients with high LAG‐3 expression were found to have a higher median survival rate, although there was no difference in overall survival (Figure 1).

### 3.2 Expression of LAG‐3 in NSCLC cell lines

**Figure 2:**
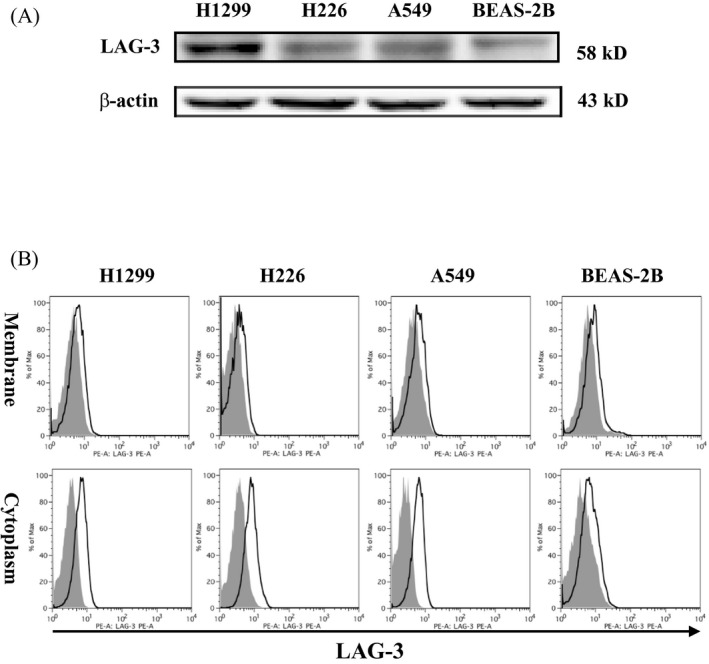
Ectopic expression of LAG‐3 in NSCLC cells. A, LAG‐3 is expressed in the H226, H1299, A549 lung cancer cell lines, and BEAS‐2B lung epithelial cells. Cells were collected from conventional cultures, and the protein levels were measured by Western blotting of whole‐cell lysates. β‐actin was used as a loading control. B, LAG‐3 was expressed not only on the cell membrane but also in the cytoplasm. Cells were collected from conventional cultures, and LAG‐3 localization was assessed by flow cytometry

Western blot assays with H226, H1299, A549, and BEAS‐2B cells revealed that LAG‐3 was expressed in both cell lines (Figure 2A). The location of the LAG‐3 expression in these cell lines was further assessed via flow cytometry. It was found that LAG‐3 was expressed not only on the cell membrane but also in the cytoplasm (Figure 2B).

### 3.3 LAG‐3 expression level in NSCLC patients

There was a significant difference in the positive rate of LAG‐3 expression in the NSCLC surgical specimens, including 20 negative cases and 32 positive cases. The benign lesions were negative for LAG‐3 expression in 13 cases and positive in 1 case. There was a significant difference in the LAG‐3 expression levels between the NSCLC and benign lung specimens (*χ* ^2^ = 13.055, *P* = .0003). The relationship between the clinicopathological features and LAG‐3 expression level in NSCLC patients is shown in Table 1. The median age was 65 years, and 33% of the patients received postoperative adjuvant chemotherapy. Of the included patients, 29% were diagnosed with squamous cell carcinoma, 71% were diagnosed with adenocarcinoma, 31% had lymph node metastasis, 23% were stage III patients, and 44% had poor differentiation.

**Table 1:** Relationship between clinicopathological features and LAG‐3 expression in patients with NSCLC (cases)

Clinicopathological features	LAG‐3 expression		*χ* ^2^	*P*
	Negative	Positive		
Gender				
Female	10	14	0.193	.660
Male	10	18		
Pathological type				
Squamous cell carcinoma	4	11	1.239	.270
Adenocarcinoma	16	21		
T staging				
T1	14	18	0.983	.320
T2 ~ T4	6	14		
N staging				
N0	16	20	1.769	.180
N1 ~ N2	4	12		
TNM staging				
I ~ II	19	21	5.983	.010
III	1	11		
Differentiation				
High and medium differentiation	13	16	1.123	.290
Low differentiation	7	16		

### 3.4 Relationship between LAG‐3 expression and clinicopathological features of patients

**Figure 3:**
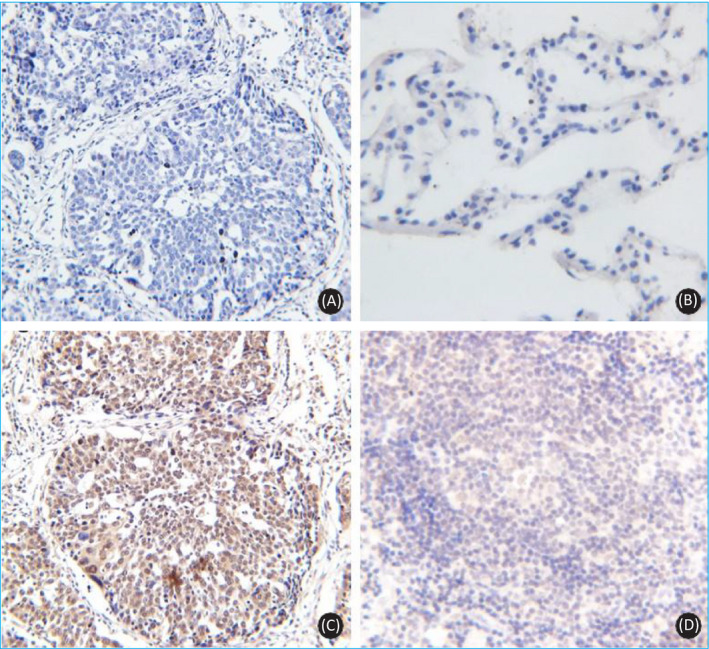
Expression of LAG‐3 in NSCLC tissue. A, NSCLC tissue specimen blank control (DAB, ×20); B, specimens of benign lung tissue lesions without LAG‐3 expression (DAB, ×40); C, NSCLC tissue specimens with very high LAG‐3 expression (DAB, ×20); D, LAG‐3 expression is visible in tumor‐infiltrating lymphocytes (DAB, ×40)

LAG‐3 was mainly expressed in lymphocytes. In this study, high ectopic LAG‐3 expression was observed in 61.5% of the NSCLC patients, and LAG‐3 expression was also observed in tumor metastatic lymph nodes. No LAG‐3 expression was observed in non‐metastatic lymph nodes. LAG‐3 expression was significantly associated with clinical stage, and LAG‐3 expression was significantly higher in stage III patients (*P* < .05). The expression level of LAG‐3 in patients with squamous cell carcinoma was higher than that in patients with adenocarcinoma, although this difference was not significant (*P* > .05). There were no significant differences in the LAG‐3 expression levels between tumor cells collected from NSCLC patients of different sex, tissue differentiation degree, T stage, or N stage (*P* > .05) (Table 1, Figure 3).

## 4 DISCUSSION

With the development of tumor immunology, increasing evidence has confirmed that the occurrence and development of tumors are caused by a variety of mechanisms. Blocking immune checkpoint molecules is considered to be an important method for preventing tumor immune escape. Although the clinical use of anti‐CTLA‐4 antibody and anti‐PD‐1/PD‐L1 antibody is considered to be a revolution in cancer therapy, most patients eventually relapse due to drug resistance; therefore, it is an urgent problem to discover new treatment schemes. Tumor‐infiltrating lymphocytes express multiple immune checkpoint molecules (eg, PD‐1, CTLA‐4, TIM‐3, LAG‐3, and TIGIT). Co‐expression of various immune checkpoint molecules can lead to T‐cell failure, which then leads to immune escape, and this effect is associated with tumor progression in patients with NSCLC.^15^ Studies have shown that blocking a single molecule leads to upregulation of the other molecules, which is one of the reasons for the low efficiency of anti‐PD‐1/PD‐L1 antibodies.^16^ LAG‐3 is a promising target, and it may be more effective than CTLA‐4 or PD‐1/PD‐L1 because anti‐LAG‐3 antibody activates effector T cells and regulatory T cells, while anti‐CTLA‐4, anti‐PD‐1, and anti‐PD‐L1 antibodies do not improve regulatory T‐cell activity.^17^ Although there are many ongoing LAG‐3‐related clinical trials, there are currently no data on LAG‐3 expression in NSCLC, and only LAG‐3 mRNA expression data are available in the existing TCGA database. LAG‐3 is mainly expressed by lymphocytes. In this study, in addition to the expression of LAG‐3 protein on tumor‐infiltrating lymphocytes, ectopic expression of LAG‐3 was also detected via immunohistochemistry in tumor tissues, but it was not expressed in benign lung tissue. Furthermore, the expression levels of LAG‐3 protein were significantly different between NSCLC and benign lung tissue samples, suggesting that LAG‐3 could be used as a biomarker for differentiating NSCLC and benign lung tissue.

There are some shortcomings in this study. Due to the limited number of samples included, further prospective studies of large NSCLC cohorts are needed to determine the critical value of positive LAG‐3 expression. However, according to the results of this study, if the ectopic expression of LAG‐3 in tumor cells can be confirmed, it may prove that LAG‐3 could have an anti‐tumor effect in addition to its role in immunological checkpoints. For example, anti‐LAG‐3 antibody therapy may directly kill tumors. Although the tumor tissue can be entirely removed via surgery in patients with early NSCLC, many patients experience recurrence shortly after surgery because of micrometastasis. The results of this study also showed that some patients with early NSCLC also exhibit LAG‐3 expression. Therefore, anti‐LAG‐3 antibody could be used to fight micrometastasis in early NSCLC patients with high LAG‐3 expression. LAG‐3 expression is significantly elevated in stage III patients, suggesting that anti‐LAG‐3 antibody might be useful as a postoperative adjuvant therapy or even as a preoperative neoadjuvant therapy, possibilities that clearly require further clinical studies.

In summary, this study found that LAG‐3 was not only expressed in tumor‐infiltrating lymphocytes in NSCLC patients, it was also ectopically expressed in tumor cells, and this expression was associated with TNM staging.
